# Co-blending modification of activated coke using pyrolusite and titanium ore for low-temperature NOx removal

**DOI:** 10.1038/s41598-020-76592-3

**Published:** 2020-11-10

**Authors:** Lin Yang, Lu Yao, Yuguo Lai, Xia Jiang, Wenju Jiang

**Affiliations:** 1 College of Architecture and Environment, Sichuan University, Chengdu, 610065 Sichuan China; 2 National Engineering Research Center for Flue Gas Desulfurization, Sichuan University, Chengdu, 610065 Sichuan China; 3 National Engineering Laboratory for Clean Technology of Leather Manufacture, Sichuan University, Chengdu, 610065 Sichuan China

**Keywords:** Pollution remediation, Chemical engineering

## Abstract

Activated coke (AC) has great potential in the field of low-temperature NO removal (DeNO_x_), especially the branch prepared by blending modification. In this study, the AC-based pyrolusite and/or titanium ore blended catalysts were prepared and applied for DeNO_x_. The results show blending pyrolusite and titanium ore promoted the catalytic performance of AC (Px@AC, Tix@AC) clearly, and the co-blending of two of them showed a synergistic effect. The (P/Ti-1/2)15@AC performed the highest NO conversion of 66.4%, improved 16.9% and 16.0% respectively compared with P15@AC and Ti15@AC. For the (P/Ti-1/2)15@AC DeNO_x_, its relative better porous structure (S_BET_ = 364 m^2^/g, V_mic_ = 0.156 cm^3^/g) makes better mass transfer and more active sites exposure, stronger surface acidity (C–O, 19.43%; C=O, 4.16%) is more favorable to the NH_3_ adsorption, and Ti, Mn and Fe formed bridge structure fasted the lactic oxygen recovery and electron transfer. The DeNO_x_ of (P/Ti-1/2)15@AC followed both the E–R and L–H mechanism, both the gaseous and adsorbed NO reacted with the activated NH_3_ due to the active sites provided by both the carbon and titanium.

## Introduction

Nitrogen oxide (NO_x_) is an important atmospheric contaminant that contributes substantially to fine particulate matter production and contributes to climate change due to its secondary transformation to nitrate^1^ and/or nitrous oxide (N_2_O, an important greenhouse gas)^2^. To reduce NO_x_ emission, therefore, is imperative for a sustainable environment and clean skies. Selective catalytic reduction (SCR) is currently the most efficient method and widely used flue gas NO_x_ removal technology^3,4^, and the key to it is the catalyst. After decades of devotion, a variety of catalysts based on different application requirements have been developed in laboratory^5–7^. Whereas only the V_2_O_5_–WO_3_(MoO_3_)/TiO_2_ series catalysts, which typically works in a high-temperature range (300–400 °C), is widely industrialized^5^. The middle- and low-temperature range NO_x_ removal in the non-ferrous metallurgy, construction materials, and coking industry et al. is still a long way to go.

Carbonaceous materials, such as activated carbon^8,9^, carbon nanotube^10^, biochar/biocarbon^11^, et al. have been attracting considerable interest in the field of air pollution control. The use of activated coke (AC)-based catalyst, in particular, has good potential for industrial application due to its relatively low cost and excellent adaptability^12^. To improve the reaction activity, integration of AC with transition metals has been studied^13,14^, and some of them observed good NH_3_-SCR performance^14–16^. However, most of the AC-based catalysts were prepared by the impregnation method, and this method can use only soluble transition metal salts with a relatively low decomposition temperature as precursor^12,17^. The catalyst preparation is complicated and high-cost, which restrain its wide application.

To simplify the preparation and reduce costs, the blending method that integrates AC preparation and catalyzation modification in one step extend the applicability of the catalyst precursor to catch great attention in the past few years. Jiang had prepared the metal oxide-blended activated carbon from walnut shells for flue gas desulfurization^18^, as well as the coal-based AC^13,19^, and the recent work observed clear improvement of SCR activity of the blending-modified AC^20^. However, studies on the SCR application of the blending-modified AC is still in its infancy. Therefore, studying NO_x_ removal using the new blending-modified AC is important in integrating low-temperature flue gas desulfurization and denitrification.

Pyrolusite and titanium ore are low-cost natural minerals and mainly contain the Mn, Fe, Ti, and other trace metal elements. Previous work reported that Fe_2_O_3_ exhibits a synergistic effect with MnO_2_ to enhance the low-temperature NO_x_ removal activity of catalyst^21^. Yang et al. found that the N_2_O formation of Mn-based SCR catalyst can be effectively inhibited using the Ti–Fe structure carrier^22^. If pyrolusite and titanium ore can be used to co-blending modification of AC to prepare a highly efficient low-temperature SCR catalyst, then the preparation cost can be significantly reduced and better industrialization prospects can be ensured.

In this study, the natural pyrolusite from South Africa and titanium ore from Sichuan, China, were used to prepare the blending-modified AC for low-temperature NO_x_ removal. Based on the characterization of porosity and surface chemistry, the denitrification performance of AC modified by single pyrolusite or titanium ore, their mixture, and the interactions of the two ores were discussed. Besides, the NO_x_ removal mechanism over the composite catalyst was proposed based on the transient response analysis coupled with the variation study of surface chemistry.

## Materials and experiments

### Materials

Bituminous coal and 1/3 coking coal from Shanxi Province, China, were used as carbon sources to prepare the new blending-modified AC catalyst. Elemental analysis (EA 3000, Elemental, Italy) showed that bituminous coal consisted of (wt%) C = 73.78, H = 9.69, N = 2.29, S = 1.76, and O = 14.10, the 1/3 coking coal consisted of (wt%) C = 74.23, H = 4.83, N = 2.08, S = 0.47, and O = 17.39. The binder used was coal tar from Sichuan Coal and Coking Group Co., Ltd. and contained (wt. %) C = 80.08, N = 1.20, H = 3.91, S = 0.75, and O = 14.06. The pyrolusite from South Africa and titanium ore from Panzhihua (China) were used as precursors to prepare the AC catalyst. Table 1 lists the ingredient analysis based on X-ray fluorescence (XRF-1800, Shimadzu, JP).

**Table 1:** Elemental analysis of the used pyrolusite and titanium ore using XRF (%).

Mineral name	Ingredients proportion							
	Mn	Fe	Ti	Al	Ca	Mg	Si	S
pyrolusite	33.20	6.13	/	0.22	8.18	1.64	2.43	0.03
Titanium ore	/	21.73	20.76	0.48	0.65	2.86	2.77	0.69

### Preparation of ACs

The bituminous coal and 1/3 coking coal mixture at a 7:3 weight ratio was used as the carbon source of AC, and the blending modification method that integrates the preparation and modification in one step was used. Coal powder was passed through a 200-mesh screen and mixed homogeneously in a kneading machine (10 min). Approximately 10.0 wt% of water and 42.0 wt% of coal tar were then successively introduced through continuous agitation. When the mixture showed a metallic luster for approximately 30 min, it was transferred to a hydraulic machine barrel and extruded at 10 MPa pressure to obtain a 3 mm diameter columnar semi-coke. The only difference in pyrolusite and titanium ore-blended AC preparation was that the calculated content of pyrolusite and/or titanium ore (also passed through a 200-mesh screen) was added directly to the coal mixture and stirred for at least 15 min before the water and coal tar were added.

After an overnight drying process, the semi-coke was activated, followed by continuous carbonization–activation process. The whole activation process involves pre-oxidation, carbonization, and steam activation at 270, 600, and 900 °C for 60, 40, and 60 min, respectively. The air was used for pre-oxidation, water was introduced during the activation at a flow rate of 0.5 mL/g·h to form steam, and other moments were run in N_2_. The tube furnace was cooled naturally at room temperature in nitrogen. The pyrolusite- and titanium-blended ACs are presented as Px@AC and Tix@AC, respectively, and the combination of the two-ore co-modified ACs is referred to as (P/Ti-a/b)x@AC. P represents pyrolusite, Ti corresponds to the titanium ore, a/b is the combined weight ratio of P and Ti, and x is the total loaded weight percentage of the carbon source.

### Characterizations

The pore structure and surface area were characterized using ASAP 2460 (Micromeritics, USA) at − 196 °C after 8 h degas at 250 °C. The nitrogen adsorbed at 0.995 relative pressure was used to calculate the total pore volume. The surface area was calculated using the Brunauer–Emmett–Teller model. The micropore volume was calculated using the t-plot model, and the mesopore distribution was obtained using the Barrett–Joyner–Halenda method. X-ray diffraction analysis was performed using an X-Pert Pro MPD diffractometer (Panalytical Co., Ltd., NLD) with Cu *Kα* radiation (30 V and 20 A) from 10° to 80°. Fourier Transform Infrared Spectroscopy (FTIR) was obtained by a Nicolet 6700 infrared spectrometer (Thermo Scientific, USA) in the range of 4000–400 cm^−1^. X-ray photoelectron spectroscopy (XPS) characterization was performed by a PHI 5000 XPS measurements (ESCA Microprobe, JP) using a monochromatic Al *Kα* X-ray source (1486.6 eV).

### Denitrification performance test

The NH_3_-SCR of ACs was conducted in a lab-scale fixed-bed evaluation system. The total flow rate of the simulated flue gas was 500 mL/min, which contained 500 ppm NO and 500 ppm NH_3_, 5.0% O_2_, and balanced with N_2_. The corresponding space velocity and reaction temperature were 1000 h^−1^ and 150 °C, respectively. The inlet and outlet NO concentrations were continuously monitored using an online flue gas analyzer (Gasboard-3000, China). Given the adsorption property of AC, NH_3_ was replaced with N_2_ in the initial stage until the outlet number of NO was almost the same as that of the inlet. After NH_3_ was introduced to the simulated gas, the denitrification test began. The NO removal performance of AC was expressed by the NO conversion and calculated based on Eq. 1, where *η* is NO conversion (%), and C_in_ and C_out_ are the inlet and outlet NO concentrations (ppm), respectively.

1 $$\eta = \frac{{C_{in} - C_{out} }}{{C_{in} }} \times 100\%$$

## Results and discussion

### Catalytic performance of AC

**Figure 1:**
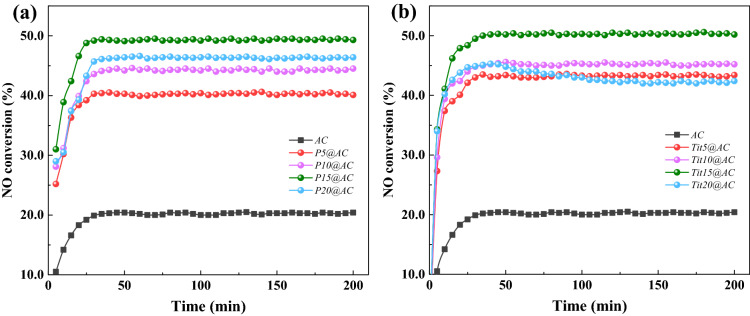
NH_3_-SCR performance of (**a**) Px@AC and (**b**) Tix@AC.

The NH_3_-SCR performance of the prepared three different AC-based catalysts is showing in Fig. 1. The NO conversion of virgin AC was only approximately 20.4%, and all the blending-modified ACs showed clear improvement. As is shown in Fig. 1a, blending 5.0 wt% of pyrolusite increased the NO removal efficiency of P5@AC to 40.5%, it was twice that of virgin AC. As the blending content gradually increased, the SCR performance of Px@AC first increased and then relatively decreased. P15@AC showed the highest NO conversion at 49.5%. Beyond this addition, the decrease in NO conversion may be attributed to the pore blockage because greater ash and metal content were easily agglomerated under high-temperature conditions^23^. The NO removal activity of Tix@AC showed a similar variation as Px@AC, as shown in Fig. 1b. The best NH_3_-SCR performance of approximately 50.5% at a 15.0 wt% blending ratio, slightly higher than that of P15@AC. The improvement of the denitrification performance indicates the suitability of pyrolusite and titanium ore as precursors to prepare the AC-based SCR catalyst. The SCR performance of Px@AC in this study was relatively lower than that reported in previous work^20^, this could be attributed to the different pyrolusite precursors used, which had a different proportion of ingredients; we advance this point in this study. The preparation process also had some differences, which affected the activity of AC through surface chemistry variation, such as the functional groups and metal active sites. Additional details of this issue are still being determined.

**Figure 2:**
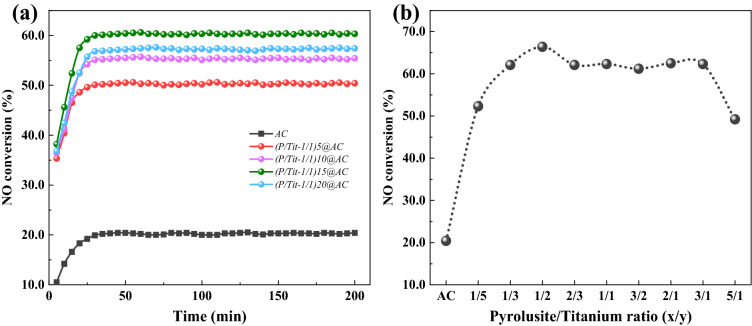
The NH_3_-SCR performance of (**a**) (P/Ti-a/b)x@AC and (**b**) (P/Ti-a/b)15@AC.

Figure 2 shows the co-blended (P/Ti-a/b)x@AC to investigate the interaction between the two different minerals. Figure 2a shows the NO removal performance of (P/Ti-1/1)x@AC at a different total dosage of pyrolusite and titanium ore. The (P/Ti-1/1)x@ACs are performing a better denitrification activity than AC, Px@AC, and Tix@AC. When the total dosage was 5.0 wt%, the co-blended sample at the pyrolusite/titanium ore proportion ((P/Ti-1/1)5@ACs) of 1/1 achieved comparable activity to the P15@AC and Ti15@AC. The catalytic performance of (P/Ti-1/1)x@AC gradually strengthened as the blending amount gradually increased, and the (P/Ti-1/1)15@AC showed the best performance at 1/1 mixing ratio, with the NO removal efficiency increasing to 60.5%. Kept the blending ratio at 15.0 wt% constant, the influence of blending proportions of pyrolusite and titanium ore was also investigated, and their NH_3_-SCR performance is shown in Fig. 2b. The denitrification performance of co-blended (P/Ti-a/b)15@AC improved when the pyrolusite/titanium ore proportion changed from 1/5 to 1/2, the highest NO conversion of 66.4% was obtained when the pyrolusite/titanium ore proportion (a/b) was 1/2 ((P/Ti-1/2)15@AC). Increasing pyrolusite content, the activity of (P/Ti-a/b)15@AC first showed a reasonable decrease and remained relatively stable in the range of 61.2%–62.3% until the a/b was 3/1. Compared with the Px@AC and Tix@AC, the improved NO removal of (P/Ti-a/b)x@AC (a/b <  = 3/1) indicates that a synergistic reaction occurred between the metal oxides in the two minerals, and the optimum proportion of the two minerals was 1/2, which corresponded to the best catalytic performance. The N_2_ selectivity test of (P/Ti-1/2)15@AC shows only ~ 25 ± 3 ppm of N_2_O detected in the outlet flue gas, corresponding to ~ 91.8% of N_2_ selectivity.

### Characterization of AC

#### Porosity of the AC

Textual properties of the (P/Ti-a/b)x@AC samples were characterized using N_2_ adsorption, and the results are listed in Table 2. The (P/Ti-a/b)x@AC samples were still typically microporous material, their V_mic_/V_tot_ are higher than 70.0%. The porosity of the (P/Ti-1/1)x@AC are worse than the virgin AC. The increased loading content showed a limited effect on the S_BET_ when the blending amount within 15.0 wt%, varying in the range from 330 to 348 m^2^/g. The low S_BET_ can be attributed to the greater ash content after the introduction of the mineral, and the metal oxides can also strengthen the agglomeration process during the carbonization–activation process^24,25^. Keep the total blending content of the two ores constant (15.0 wt%), the S_BET_ and pore volume were similar to the characteristic of “mountain” with the increased proportion of pyrolusite. The (P/Ti-1/2)15@AC had the best textual properties of 364 m^2^/g of S_BET_ and 0.192 cm^3^/g of V_tot_. S_BET_ increased as the pyrolusite increased from 1/5 to 1/2, thus indicating that pyrolusite could certainly improve the pore structure of AC during water steam activation. This result is consistent with the previous study^24^. Figure 2a shows the catalytic performance of (P/Ti-1/1)x@AC strengthened gradually when the blending ratio changed from 5.0 wt% to 15.0 wt%. This finding indicates that the textural properties of the catalysts were not the pacing factor to determine the catalytic activity, and the surface metal oxides and functional groups could be the principal in determining NO removal^26^. The S_BET_ and pore volume of the (P/Ti-a/b)15@AC sample further confirmed this point. As the pyrolusite/titanium ore ratio (a/b) varied from 1/5 to 1/2, the S_BET_ and pore volume of (P/Ti-a/b)15@AC increased after the NH_3_-SCR performance improved. As the pyrolusite percentage increased, especially when the a/b was in the range from 2/3 to 2/1, the (P/Ti-a/b)15@AC showed relatively poor activity (Fig. 2b) despite its comparable porosity (Table 2).

**Table 2:** Textural property of the prepared ACs.

Samples	S_BET_ (m^2^/g)	V_tot_ (cm^3^/g)	V_mic_ (cm^3^/g)	V_mes_ (cm^3^/g)	AD (nm)
AC	415	0.224	0.170	0.038	2.20
(P/Ti-1/1)5@AC	330	0.182	0.145	0.012	2.20
(P/Ti-1/1)10@AC	346	0.185	0.143	0.028	2.10
(P/Ti-1/1)15@AC	338	0.185	0.138	0.033	2.20
(P/Ti-1/1)20@AC	307	0.172	0.122	0.037	2.20
(P/Ti-1/5)15@AC	332	0.185	0.137	0.045	2.30
(P/Ti-1/3)15@AC	347	0.191	0.147	0.025	2.20
(P/Ti-1/2)15@AC	364	0.192	0.156	0.024	2.10
(P/Ti-2/3)15@AC	344	0.187	0.142	0.031	2.20
(P/Ti-3/2)15@AC	337	0.185	0.138	0.045	2.30
(P/Ti-2/1)15@AC	363	0.185	0.155	0.015	2.00
(P/Ti-3/1)15@AC	346	0.192	0.141	0.037	2.20
(P/Ti-5/1)15@AC	328	0.185	0.136	0.047	2.30

S_BET_: the BET surface area; V_tot_: the total pore volume; V_mic_: the micropore volume; V_mes_: the mesopore volume; and AD: the average pore diameter.

#### Surface functional groups

**Figure 3:**
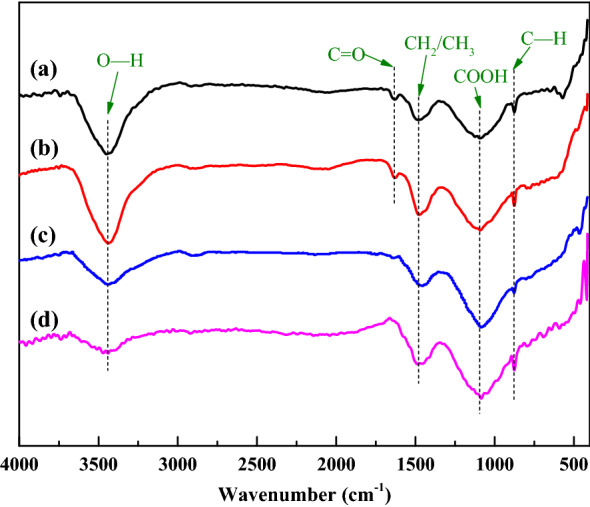
The FTIR spectra of AC (**a**, **b**) and (P/Ti-1/2)15@AC (**c**, **d**) before (**a**, **c**) and after (**b**, **d**) the denitrification application.

FTIR analysis was applied to analyze the surface functional groups of the AC and (P/Ti-1/2)15@AC. As is shown in Fig. 3, both samples show almost similar FTIR spectra, differences showed in their intensities and slightly shift suggesting very limited changes of the surface chemistry^25^. For both the AC and (P/Ti-1/2)15@AC, their spectra assigned the absorption band to the O–H stretching vibration (approximately 3400 cm^−1^)^27^, CH_2_ and CH_3_ (1460 cm^−1^)^28^, COOH (approximately 1090 cm^−1^), and C–H (at 875 cm^−1^)^29^, respectively. Be differently, AC showed adsorption band at 1640–1630 cm^−1^, which belongs to the moisture peaks overlapped with C=C and C=O stretching in phenylpropanoid side chains, and aromatic skeleton vibration^30^. The disappearance of the 1640–1630 cm^−1^ band on (P/Ti-1/2)15@AC could be due to the bridging interactions of the blended micro metal oxide particles to the surface of AC^31^. The intensity of COOH was enhanced by the pyrolusite and titanium ore co-blending, indicating a stronger surface acidity of (P/Ti-1/2)15@AC, coupling with the loss of C=O located at 1640–1630 cm^−1^, the surface NH_3_ adsorption could be fasted to improve the SCR performance of (P/Ti-1/2)15@AC.

**Figure 4:**
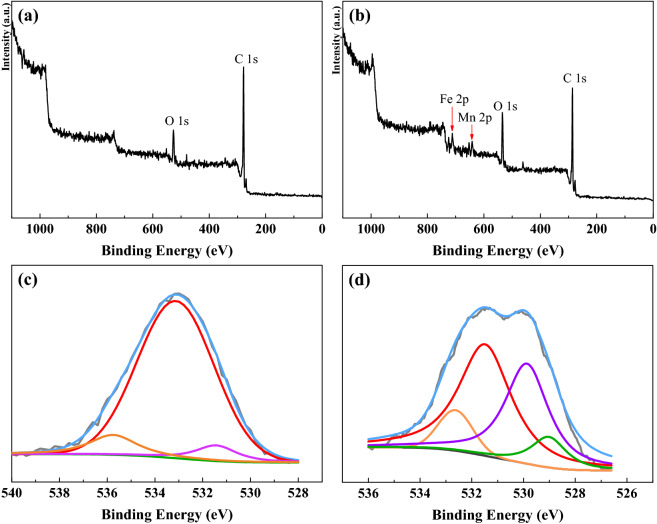
The full XPS spectrum of AC (**a**) and (P/Ti-1/2)15@AC (**b**), the O 1*s* spectra of AC (**c**), and (P/Ti-1/2)15@AC (**d**).

The XPS characterization was applied to gain insight into the nature of surface functional groups. The full XPS spectrum of AC and (P/Ti-1/2)15@AC is shown in Fig. 4a,b, and the surface composition is based on it listed in Table 3. The (P/Ti-1/2)15@AC had high surface oxygen content, it was 17.11%, 4.58% higher than AC (12.53%), which indicates the participation of blended metal oxides during steam activation helps the AC reserve surface oxygen components. Table 4 shows the functional group distribution based on the C 1*s* high deconvolution spectrum. The C–C of (P/Ti-1/2)15@AC is 4.42% less than that of virgin AC. Additional oxygen functional groups were observed, and the relative contents of C–O, C=O, and π–π* increased by 3.32%, 0.78%, and 0.40% compared with the virgin AC when (P/Ti-1/2)15@AC was treated with strong surface acidity^8^. The O 1*s* deconvolution spectra are showing in Fig. 4c, d. Both AC and (P/Ti-1/2)15@AC have three energy peaks. They were Peak I (assigned at 530.4–531.0 eV) belongs to C=O groups in esters, carbonyl, and quinone, Peak II (centered at 532.4–533.1 eV) corresponds to C–OH and/or C–O–C groups in esters, amides, and others, and Peak III (located at 533.6–535.6 eV) corresponds to chemisorbed oxygen and/or water. Given the blended metal oxides, (P/Ti-1/2)15@AC had another peak centered at 529.2–530.3 eV, which was mainly attributed to the lattice oxygen^32^. The increased lattice oxygen of (P/Ti-1/2)15@AC is consistent with its improved NO removal performance and plays an important role in promoting the formation of nitrate species by participating in the NO oxidation, thus further hastening the NH_3_-SCR^33^. Thus, the chemical properties of AC, such as oxygen functional groups and lattice oxygen, were more important factors than the physical properties, which is consistent with the previous results^34^. The consensus is that the oxygen vacancy and lattice could facilitate the oxidation of NO to NO_2_ and further accelerate the ‘fast SCR’ process^35^.

**Table 3:** Surface atomic content of C and O based on XPS.

Samples	Atomic concentration (%)		Atomic ratio (O/C, %)
	C	O	
AC	87.16	12.53	14.38
DNOAC*	82.30	17.19	20.89
(P/Tit-1/2)15@AC	80.85	17.11	21.16
DNO(P/Tit-1/2)15@AC*	78.52	18.05	22.99

*D_NO_AC and D_NO_(P/Tit-1/2)15@AC were the AC and (P/Tit-1/2)15@AC after NO removal.

**Table 4:** Relative content of the surface functional groups before and after NO removal^28^.

Groups	Binding energy (eV)	Relative content (%)			
		AC	D_NO_AC	(P/Ti-1/2)15@AC	D_NO_(P/Ti-1/2)15@AC
C–C	284.6	76.51	74.76	72.09	70.48
C–O	285.1–286.1	16.11	17.85	19.43	22.43
C=O	286.8–288.3	3.48	3.62	4.16	4.08
π–π*	288.6–291.5	3.92	3.77	4.32	3.01

#### Metallic chemistry characterization

**Figure 5:**
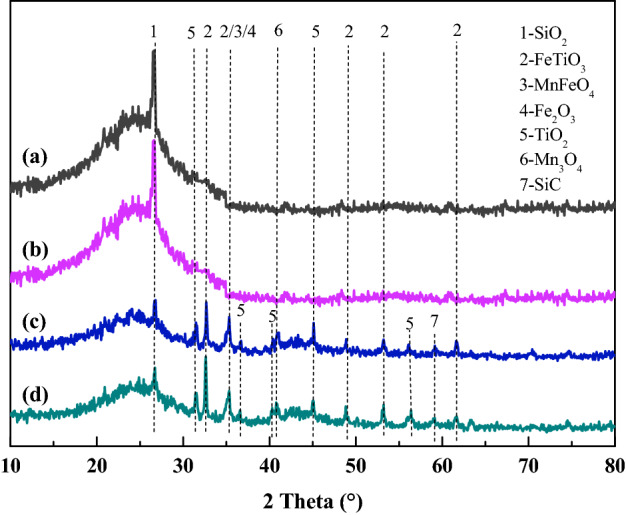
The XRD analysis of AC (**a**, **b**) and (P/Ti-1/2)15@AC (**c**, **d**) before (**a**, **c**) and after (**b**, **d**) the catalytic NO removal.

Figure 5 shows the XRD patterns of the AC and (P/Ti-1/2)15@AC before and after NO removal. For the AC sample, only one clear SiO_2_ peak at 2θ = 26.71° was detected without considering the carbon base. For the (P/Ti-1/2)15@AC, the SiO_2_ peak was weakened, and the peaks located at 32.48°, 35.25°, 48.70°, 53.10°, and 61.54° matched well with FeTiO_3_ (JCPD 29-0733). The MnFeO_4_ at 2θ = 34.98° (JCPD 29-0733) and Fe_2_O_3_ at 2θ = 35.42° (JCPD 99-0073) were also detected, but they were covered or bonded to the FeTiO_3_ peak at 35.25°. Besides, there was a peak ascribed to SiC at 2θ = 59.1° was detected, indicating the potential catalysis of the metallic of carbon and SiO_2_^36^. The weakened peaks of TiO_2_ at 2θ = 36.6°, 40.3°, 56.4°^37^ could confirm the formation of bimetallic structure. The detected FeTiO_3_ and MnFeO_4_ indicated that the blended Fe, Mn, and Ti can form a bimetal or trimetal bridge structure on the surface of AC. The consumed lattice oxygen of the bridge-structured multi-metal oxides can be replenished easily by gaseous oxygen, which improves the catalytic removal of NO significantly^22,38^.

**Figure 6:**
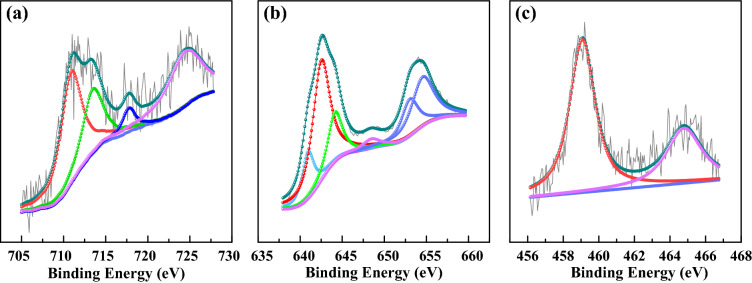
The Fe 2*p* (**a**), Mn 2*p* (**b**) and Ti 2*p* (**c**) spectra of (P/Ti-1/2)15@AC.

Figure 6 displays the Fe 2*p*, Mn 2*p*, and Ti 2*p* XPS spectrum of the new (P/Ti-1/2)15@AC. The Fe 2*p*_3/2_ profile has three overlapping peaks at 710.8, 713.1, and 718.7 eV, which are assigned to the Fe^3+^ bonded with hydroxyl (OH)^39,40^. The Mn 2*p*_3/2_ profiles have three overlapping peaks corresponding to Mn^4+^ (644.1 eV), Mn^3+^ (642.5 eV), and Mn^2+^ (640.9 eV)^12,40^. Two peaks at 464.8 eV (Ti 2*p*_3/2_) and 459.1 eV (Ti 2*p*_1/2_) were obtained with a 5.7 eV spin-orbital-splitting belong to the rutile TiO_2_^41^. The slight shift of binding energy compared with their standard curves in the Fe 2*p* and Mn 2*p* spectrums confirm the incorporation of different metal oxides. It was reported that manganese oxides generally contain varieties of labile oxygen, which plays important role in the low-temperature catalytic removal of NO with NH_3_^42^. Figures 4b and 6 show the labile oxygen and manganese oxidation states (Mn^2+^, Mn^3+^, Mn^4+^) coexisted on the surface of (P/Ti-1/2)15@AC. The transformation of coordinated NH_3_ to NH_2_ on Mn^3+^ species initiated the SCR reaction^43^. The iron oxides can promote the Mn-based catalyst due to its high activity and thermal stability. The redox cycles of Fe^3+^ ↔ Fe^2+^ and Mn^4+^ ↔ Mn^3+^ ↔ Mn^2+^ work as a multifunctional electron transfer bridge and release surface oxygen during the catalytic NO removal^22^, which also makes the catalytic reaction continuable. The improved catalytic performance of (P/Ti-a/b)15@AC is also contributed by the TiO_2_ species, which shows acidic property and greatly improves the surface NH_3_ adsorption during flue gas denitrification^44^.

#### Surface chemistry after NO removal

The surface chemistry of the NH_3_-SCR used AC and (P/Ti-1/2)15@AC was studied to obtain additional information. Figure 3b shows that the O–H intensity at 3400 cm^−1^ of AC strengthened after the NO removal reaction, whereas the (P/Ti-1/2)15@AC showed almost no change. This finding indicates that the blended metal oxides can improve the surface hydrophobicity of the catalyst, and the H_2_O generated by the reduction removal of NO can be easily carried off at low temperature. In addition, the characterization peaks at 1460 and 875 cm^−1^ for both AC and (P/Ti-1/2)15@AC were intensified possibly because of the adsorbed NH_3_ dehydrogenation process (Fig. 3b, d), which was reported as the controlling step of denitrification^45,46^. On the basis of the XPS results, the additional surface oxygen of the sample after the SCR reaction increased by 4.66% and 0.94% of AC and (P/Ti-1/2)15@AC (Table 3). Both the AC and (P/Ti-1/2)15@AC had a high C–O content after NO removal because of the surface oxygen adsorption during the reaction process^9^. The π–π* bond of both samples were consumed somehow, which can be attributed to the participation of π bonds in the NH_3_–NO–O_2_ reaction^47^. Consistent with the FTIR, the C=O changed only slightly. No clear difference was detected in the metallic chemistry according to the XRD analysis. This result is consistent with the foregoing conclusion and indicates that the multi-metal structure can perform a stable catalytic activity.

### NO removal mechanism

**Figure 7:**
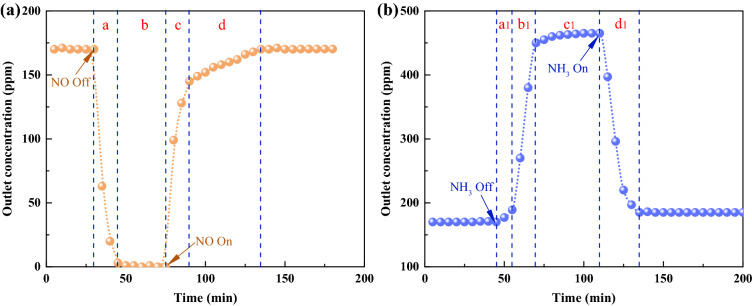
Transient selective catalytic reduction experiments of (P/Ti-1/2)15@AC with step feed of NO (**a**) and NH_3_ (**b**) at 150 °C.

Transient response method (TRM) experiments were conducted to analyze the dynamics of the NH_3_-SCR process of (P/Ti-1/2)15@AC^48^. Figure 7a shows that the denitrification process responded rapidly with NO, and the outlet NO reduced in 10 min from steady-state to 0 ppm when the inlet NO was completely converted (stage *a*). When the NO was fed again, however, the outlet NO had a slowly increasing segment (stage *d*) after a sharp, short increase. This phenomenon can be attributed to the adsorption of NO during the reduction process. The step feed of NO indicates that both the gaseous and absorbed NO participated in selective catalytic removal on the (P/Ti-1/2)15@AC surface, but the adsorbed NO is the main part. Figure 7b shows the NH_3_ TRM experiment, and the outlet NO showed a two-step increase after the NH_3_ was turned off. Within a few minutes (approximately 10 min) after NH_3_ was converted from feedback (stage *a1*), the NO concentration of outlet gas increased slowly from 170 to 189 ppm, which indicates that some adsorbed NH_3_ can participate in the SCR reaction^22^. With the NH_3_ consumed, the outlet NO gradually increased (stage *b1*) and remained relatively stable at approximately 465 ppm (stage *c1*). The 7.0% NO removal without NH_3_ supply can be due to the surface adsorption and oxidation of NO to form some nitrite and/or nitrate^49^. After the NH_3_ supply is fed again, the NO conversion showed almost a linear recovery in 25 min (stage *d1*). The NO variation with the step-feed NH_3_ indicates that the NH_3_ must be adsorbed first to participate in the SCR reaction.

**Figure 8:**
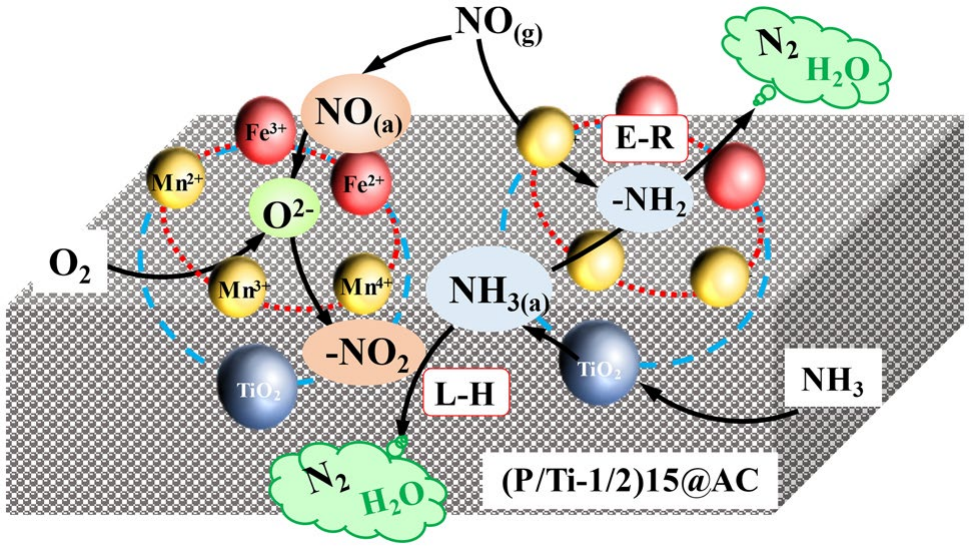
The low-temperature NO removal mechanism over the (P/Ti-1/2)15@AC composite catalyst.

On the basis of the results of this work and previous reports, the Eley–Rideal (E–R) and Langmuir–Hinshelwood (L–H) mechanisms coexist in the NH_3_-SCR reaction over (P/Ti-1/2)15@AC^45,50,51^. Both the gaseous and adsorbed NO reacted to the surface adsorbed NH_3_ due to the carbon and titanium composite structure. In the L–H mechanism pathway, the gaseous NH_3_ and NO first adsorbed on the (P/Ti-1/2)15@AC surface (Eqs. 2, 3). The adsorbed NO was oxidized to nitrite by the lactic and surface-active oxygen (Eq. 4). The NH_3(ads)_ then reacted with the nitrite to form the NH_3_NO_2_ intermediates (Eq. 5) and finally produce the N_2_ and H_2_O products through a disproportionate reaction (Eq. 5). In the L–H pathway, the NH_3(ads)_ can be activated by the metal oxide (MnO_2_) to form the –NH_2_ (Eq. 6) and can be reacted with the gaseous NO directly via the E–R mechanism (Eq. 7). The preliminarily established entire NO removal mechanism over the (P/Ti-1/2)15@AC composite catalyst is illustrated in Fig. 8.

2 $$NO_{(g)} \rightarrow NO_{(ads)}$$

3 $$NH_{3(g)} \rightarrow NH_{3(ads)}$$

4 $$NO_{(ads)} + M{ - }O* \rightarrow M{ - }O{ - }NO$$

5 $$NH_{3(ads)} + M{ - }O{ - }NO \rightarrow M{ - }O{ - }NO{ - }NH_{3} \rightarrow M{ - }OH + N_{2({\rm g})} + H_{2} O_{(g)} \quad ({\text{L}{-}\text{H}})$$

6 $$NH_{3(ads)} + M{ - }O* \rightarrow {-}NH_{2} + M{-}OH$$

7 $${ - }NH_{2} + NO_{(g)} \rightarrow NH_{2} NO \rightarrow N_{2({\rm g})} + H_{2} O_{(g)} \left( {{\text{E}} {-} {\text{R}}} \right)$$

Table 5 listed some NO removal performance comparison with those of literature that have been reported. At 150 °C, the NO conversion of (P/Ti-1/2)15@AC (in the current study) was 66.4%, which is clearly higher than the reported V_2_O_5_/AC, Cu/AC-IM, Ce/AC-CNTs et al. catalyst. The Cu/AC-N-IM performed the closest NO conversion is still 9.2% lower than (P/Ti-1/2)15@AC. We know there are many catalysts in related work performed a better NO removal efficiency, while most of them were prepared by the impregnation method as the introduction mentioned (as well the catalysts listed in Table 5), their preparation cost and stability of catalyst were their momentous limitations, especially in industrial application.

**Table 5:** Comparison of NO removal performance of various carbon-based catalysts.

Samples	Temperature (°C)	NO conversion (%)	References
(P/Ti-1/2)15@AC	150	66.4	This work
V_2_O_5_/AC	150	30	52
Mn—CeOx/biochar (COA)	180	42.9	53
Fe–Cu–O/CNTs–TiO_2_	250	12	54
Cu/AC-IM	275	49.6	55
Cu/AC-N-IM	275	57.2	55
Ce/AC-CNTs	150	41	10

## Conclusions

In this study, the AC catalysts for low-temperature NO removal using pyrolusite and titanium ore precursor were prepared. The denitrification application shows that pyrolusite and titanium ore blending could promote the catalytic performance clearly because of the more favorable structural properties, surface acidity, and labile oxygen and catalysis of transition metal oxide of AC. Compared with the monomineral modification, the pyrolusite and titanium ore co-blending enhanced the NO removal furtherly. The (P/Ti-1/2)15@AC had the highest NO conversion of 66.4%, improved 16.9%, and 16.0% compared with P15@AC and Ti15@AC. The Ti, Mn, and Fe were good bonded and formed bridge structure, which fasted the lactic oxygen recovery and electron transfer, as well the NH_3_ adsorption to have a better NO removal performance. The NO removal of (P/Ti-1/2)15@AC followed both the Eley–Rideal and Langmuir–Hinshelwood mechanisms, the gaseous and adsorbed NO reacted simultaneously with the activated NH_3_ on the surface due to the carbon and titanium composite structure.
